# Effect of open versus video-assisted thoracoscopy on perioperative outcomes and survival for cases of thymic carcinomas and thymic neuroendocrine tumors

**DOI:** 10.1186/s12957-023-03210-7

**Published:** 2023-10-16

**Authors:** Gaiyan Li, Hao Chang, Zhuangzhuang Wang, Dongjie He, Lin Qu, Qiuju Shao, Qiming Wang

**Affiliations:** 1 Department of Radiotherapy Oncology, Second Affiliated Hospital of Air Force Military Medical University, Xi’an, China; 2 Department of Neurosurgery, Xi’an Daxing Hospital, Xi’an, China; 3 Department of Thoracic Surgery, Second Affiliated Hospital of Air Force Military Medical University, Xi’an, China

**Keywords:** Thymic carcinomas, Thymic neuroendocrine tumors, Video-assisted thoracoscopic surgery, Open chest, Survival, Perioperative indicators

## Abstract

**Background:**

The oncology-related indices between open and video-assisted thoracoscopic surgery (VATS) procedures for thymic carcinomas (TCs) and thymic neuroendocrine tumors (TNETs) remain unclear.

**Methods:**

Propensity score matching (PSM) and multivariate Cox proportional risk models were used to evaluate the perioperative outcomes and survival rates of patients undergoing open and VATS for TCs and TNETs at the Second Affiliated Hospital of Air Force Military Medical University Hospital, between 2009 and 2018.

**Results:**

Of the total 126 cases of TCs and TNETs, VATS treatment was used in 39 (30.9%). Advanced age and Masaoka-Koga staging were found to be independent prognostic factors for both TCs and TNETs, through a multifactorial Cox regression analysis. There was no significant difference in survival between the VATS and open groups before and after PSM; however, the VATS group had better perioperative-related indicators. There were no significant differences between the groups in terms of mortality at 30 days, mortality at 90 days, R0 resection rate, and 5-year survival rate (67.5% vs. 58.5% [*P* = 0.260] in the VATS group compared to the open group, in a PSM analysis of the 27 VATS and 27 open groups). Compared to the open group, the VATS group had a shorter length of hospital stay (13 days vs. 16 days, *P* = 0.015), a shorter level I care (0 days vs. 1 day, *P* = 0.016), and less intraoperative bleeding (50 mL vs. 300 mL, *P* < 0.001).

**Conclusions:**

In this single-center retrospective study of TCs and TNETs, survival rates were comparable between the VATS group and the open group, and the VATS group showed improved perioperative-related parameters.

**Supplementary Information:**

The online version contains supplementary material available at 10.1186/s12957-023-03210-7.

## Background

Thymic epithelial tumors (TETs) are classified pathologically as thymomas, thymic carcinomas (TCs), and thymic neuroendocrine tumors (TNETs) [1]. TCs have a low incidence [2] and have previously been reported in the literature to have an incidence of between 0.07 and 0.38/100,000/year [3, 4]. TNETs are the rarest type of TET, accounting for just 0.4% of all carcinoid tumors. Surgery plays a central role in the comprehensive treatment of patients with both early-stage and locally-advanced TETs [5, 6], with median sternal incision being the typical preferred approach. However, as surgical techniques have advanced over time, video-assisted thoracoscopic surgery (VATS) procedures are being used increasingly to treat TETs [7, 8]. However, the clinical management of TCs and TNETs remains challenging due to their rarity (which limits their characterization through prospective large-scale clinical studies), and the lack of standardized surgical treatments for these diseases [9]. There is a scarcity of data comparing thoracoscopic and open surgery for TCs and TNETs in the literature. One single-center study compared eight patients with TC who underwent either VATS or open thymectomy procedures [10]. Another study used data from the National Cancer Database (NCDB) to compare short-term results and overall survival following open versus minimally invasive thymectomy (MIT) procedures for Masaoka-Koga stage I–III TCs. It is worth noting, however, that only 22 of the patients in that study underwent VATS [11]. Gu et al. explored the impacts of different surgical methods for locally advanced resectable TETs on patient prognoses, including 23 minimally invasive TC and TNET tumors [12]. It is currently difficult to answer specific questions regarding the optimal surgical method (i.e., VATS vs. open) for TCs and TNETs. The relevant evidence is unfortunately not yet conclusive, so more research is needed to address this clinical question. This study aimed to compare the effects of open vs VATS surgeries on perioperative outcomes and survival in cases of TCs and TNETs.

## Methods

### Study design

We pulled surgical records from 193 patients who were diagnosed with TC or TNET, for which they were treated surgically at the Second Affiliated Hospital of Air Force Military Medical University Hospital between January 2009 and December 2018. Of these, 33 were excluded, leaving 160 patients. The exclusion criteria were as follows: (1) Patients who only underwent thoracoscopic pathological biopsy procedures (12 cases); (2) patients under the age of 18 years (3 cases); (3) patients with prior histories of malignancies (11 cases); and (4) patients for whom other relevant clinical information was missing (7 cases). After a first phase of electronic case visit inquiries, a second phase of follow-ups over the telephone, and a third phase of follow-ups using short text-messages follow-up, 34 cases were lost to follow-up. As a result, 126 patients were eventually included in this study (Additional file 1: Figure 1). The primary outcome was overall survival, from the date of surgery for TC or TNET, until all causes of death or the final follow-up assessment. Perioperative-related variables represented a secondary outcome.

### Statistical analysis

TCs and TNETs of all stages were separated into two groups based on whether they were treated by VATS or open surgery, and differences between the two groups were analyzed using Pearson’s Chi-squared test, Fisher’s exact test for categorical data, and the Wilcoxon rank-sum test for continuous variables.

The data were analyzed using the real surgical method, and 13 patients with open-chest VATS were included in the open group. A multivariate Cox proportional risk model was used to compare the survival outcomes in terms of TCs and TNETs between the open and VATS groups.

To account for baseline variables between the open and VATS groups, we used propensity score matching (PSM) analysis to compare perioperative outcomes and overall survival between the two surgical techniques. The PSM therefore represented the probability of receiving open vs. VATS treatment allocation based on age, gender, Eastern Cooperative Oncology Group (ECOG) score, tumor size, malignancy stage, and type of pathology, as determined by analyzing these factors through a logistic regression model. All factors used for the model were found to be clinically significant. The best matching pair was then found using a closest-neighbor matching method, with a caliper value of 0.02. Following PSM, standardized differences were used to measure the balance between pairings. Kaplan–Meier analysis was used to analyze the overall survival in both groups, both before and after PSM. The perioperative indexes and short-term survival rates were also compared between the two groups. In order to control for the confounding effect of late staging on both surgical modalities, the same study was done on patients with stage I-IIIA TCs and TNETs.

## Results

### Population baseline characteristics

The median age of the overall cohort was 56 years, with 68.3% of the patients being male. The majority of the population had some sort of health insurance. Chest pain was the main symptom in 46 cases, accounting for 36.5% of the total population. There was only one instance of *myasthenia gravis* verified by a neostigmine test and electromyography, whose pathological type was small-cell thymic carcinoma. In 88.1% of the patients, the ECOG scores were between 0 and 1; in 54.0%, the age-adjusted Charlson comorbidity index (ACCI) scores were also between 0 and 1. Preoperative pulmonary function tests were performed on each patient, and 57.1% of the cohort were found to have generally normal lung functions. The median TET size was 5.5 cm, and the Masaoka-Koga staging of the overall cohort was 30.2% for stage I–II, 52.4% for stage III, and 17.5% for stage IV. TCs were present in 78.6% of the cohort, with 83 (65.9%) having squamous cell carcinomas. In addition, 21.5% of the patients with TNETs had four small-cell carcinomas. In terms of adjuvant therapy, 2 (1.6%) patients received neoadjuvant chemotherapy, 96 (76.2%) patients received postoperative chemotherapy, 3 (2.4%) patients received neoadjuvant radiotherapy, 90 (71.4%) patients received adjuvant radiotherapy, and 12 patients received immunotherapy after recurrent metastases developed (Table 1). The surgical accesses for the whole population are detailed in Additional file 6: Table 4.

**Table 1:** Baseline characteristics of the population before and after PSM

**Variables**	**Before PSM**			**After PSM**		
	**Open (*n* = 87)**	**VATS (*n* = 39)**	***P***	**Open (*n* = 27)**	**VATS (*n* = 27)**	***P***
**Age, Median (IQR)**	56.0 (48.5, 63.0)	58.0 (47.5, 65.0)	0.714	56.0 (49.5, 64.0)	58.0 (45.5, 65.5)	0.828
**Sex, *n* (%)**			0.108			1.000
Female	32 (36.8)	8 (20.5)		5 (18.5)	5 (18.5)	
Male	55 (63.2)	31 (79.5)		22 (81.5)	22 (81.5)	
**Insurance, *n* (%)**			0.132			0.324
Resident	37 (42.5)	11 (28.2)		12 (44.4)	6 (22.2)	
Employee	23 (26.4)	18 (46.2)		7 (25.9)	12 (44.4)	
Self-financed	5 (5.7)	3 (7.7)		2 (7.4)	2 (7.4)	
Non local	22 (25.3)	7 (17.9)		6 (22.2)	7 (25.9)	
**Symptom, *n* (%)**			0.096			0.372
Physical examination	21 (24.1)	17 (43.6)		10 (37)	7 (25.9)	
Chest pain	32 (36.8)	14 (35.9)		9 (33.3)	12 (44.4)	
Respiratory symptoms	18 (20.7)	5 (12.8)		2 (7.4)	5 (18.5)	
Other compression symptoms	16 (18.4)	3 (7.7)		6 (22.2)	3 (11.1)	
**Myasthenia gravis, *n* (%)**			0.310			1.000
No	87 (100)	38 (97.4)		27 (100)	26 (96.3)	
Yes	0 (0)	1 (2.6)		0 (0)	1 (3.7)	
**ECOG, *n* (%)**			0.050			0.863
0	21 (24.1)	18 (46.2)		10 (37)	8 (29.6)	
1	55 (63.2)	17 (43.6)		14 (51.9)	15 (55.6)	
2	11 (12.6)	4 (10.3)		3 (11.1)	4 (14.8)	
**ACCI, n (%)**			0.614			0.506
0	19 (21.8)	8 (20.5)		6 (22.2)	7 (25.9)	
1	31 (35.6)	10 (25.6)		10 (37)	6 (22.2)	
2	25 (28.7)	13 (33.3)		6 (22.2)	10 (37)	
≥ 3	12 (13.8)	8 (20.5)		5 (18.5)	4 (14.8)	
**Pulmonary function, *n* (%)**			0.202			0.715
Normal	51 (58.6)	21 (53.8)		15 (55.6)	17 (63)	
Mild	21 (24.1)	15 (38.5)		7 (25.9)	8 (29.6)	
Moderate	13 (14.9)	2 (5.1)		4 (14.8)	1 (3.7)	
Severe	2 (2.3)	1 (2.6)		1 (3.7)	1 (3.7)	
**Tumor size (cm), Median (IQR)**	6.4 (4.9, 8.2)	4.3 (3.4, 5.2)	< 0.001	5.0 ± 2.0	4.9 ± 1.6	0.921
**Masaoka-Koga stage, *n* (%)**			0.031			0.666
I-IIA	5 (5.7)	7 (17.9)		3 (11.1)	5 (18.5)	
IIB	14 (16.1)	12 (30.8)		8 (29.6)	7 (25.9)	
IIIA	27 (31)	8 (20.5)		8 (29.6)	4 (14.8)	
IIIB	26 (29.9)	5 (12.8)		4 (14.8)	5 (18.5)	
IVA	1 (1.1)	1 (2.6)		0 (0.0)	0 (0.0)	
IVB	14 (16.1)	6 (15.4)		4 (14.8)	6 (22.2)	
**Pathology, *n* (%)**			0.308			0.326
Squamous carcinoma	57 (65.5)	26 (66.7)		23 (85.2)	19 (70.4)	
Adenocarcinoma	9 (10.3)	2 (5.1)				
Sarcomatoid carcinoma	4 (4.6)	0 (0)				
Epithelial myoepithelial carcinoma	1 (1.1)	0 (0)				
Small cell carcinoma	2 (2.3)	2 (5.1)		4 (14.8)	8 (29.6)	
Large cell carcinoma	0 (0)	1 (2.6)				
Carcinoid	5 (5.7)	2 (5.1)				
Atypical carcinoid	8 (9.2)	3 (7.7)				
Neuroendocrine carcinoma,NOS	1 (1.1)	3 (7.7)				
**Chemotherapy, *n* (%)**			0.238			1.000
No	16 (18.4)	12 (30.8)		9 (33.3)	8 (29.6)	
Neoadjuvant	2 (2.3)	0 (0)		18 (66.7)	19 (70.4)	
Adjuvant	69 (79.3)	27 (69.2)				
**Radiotherapy, *n* (%)**			0.680			0.773
No	22 (25.3)	11 (28.2)		8 (29.6)	10 (37)	
Neoadjuvant	3 (3.4)	0 (0)		19 (70.4)	17 (63.0)	
Adjuvant	62 (71.3)	28 (71.8)				
**Immunotherapy, *n* (%)**			0.103			1.000
No	76 (87.4)	38 (97.4)		26 (96.3)	26 (96.3)	
Yes	11 (12.6)	1 (2.6)		1 (3.7)	1 (3.7)	

**Fig. 1:**
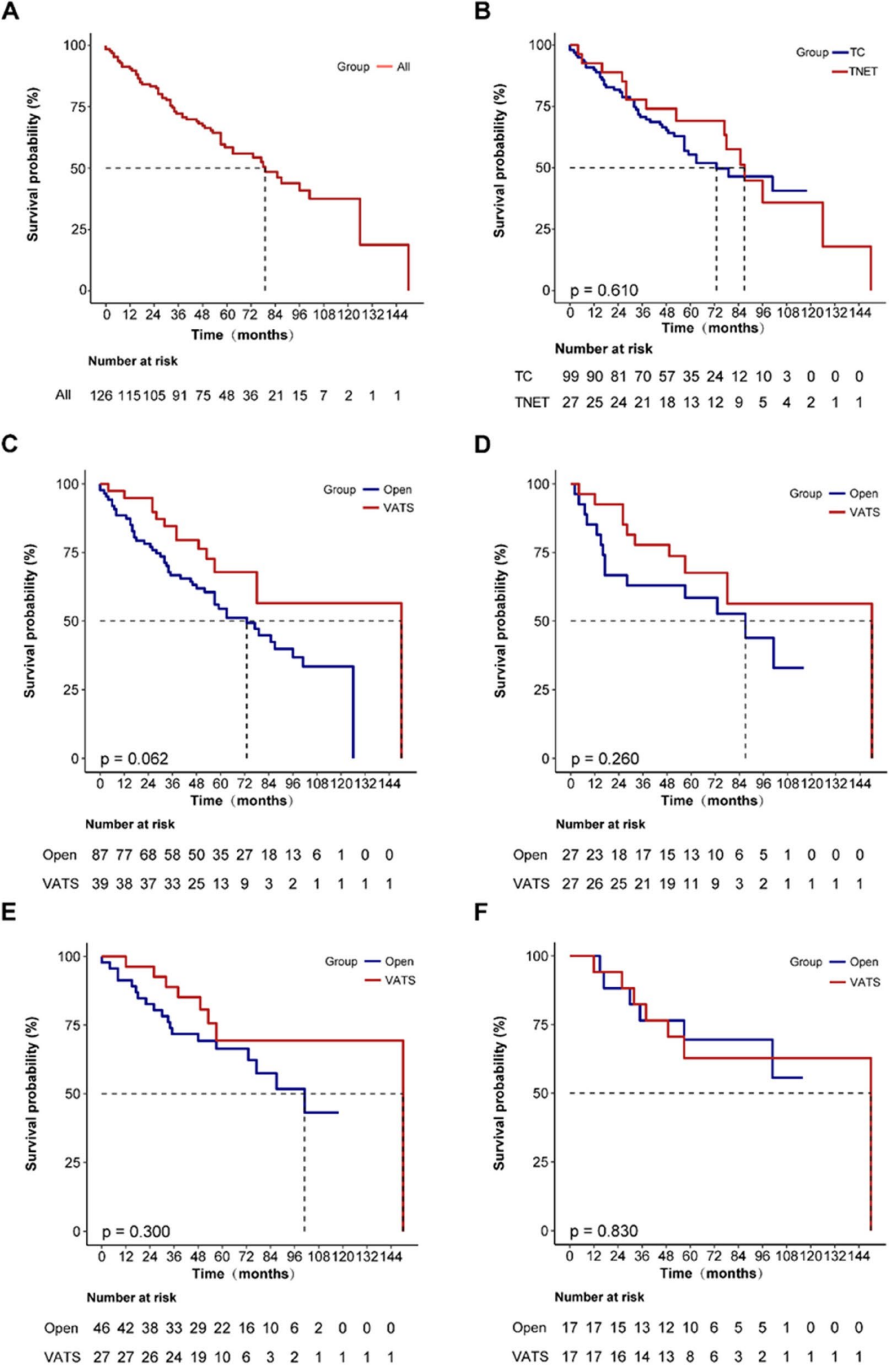
K-M survival curves (**A** entire group of people; **B** TCs and TNETs group; **C** open and VATS groups before PSM in the entire population; **D** open and VATS groups after PSM in the entire population; **E** open and VATS groups before PSM in stage I-IIIA patients; **F** open and VATS groups after PSM in stage I-IIIA patients)

The follow-up deadline was May 27, 2022. The overall cohort’s median follow-up period was 71 (95% confidence interval [CI]: 60–76) months, and the median 5-year survival rate was 58.5% (95% CI: 50–68.4%; Fig. 1A). Cases of TCs and TNETs had 5-year survival rates of 55.4% and 69.1%, respectively (*P* = 0.610; Fig. 1B).

### Trends in the use of video-assisted thoracoscopic surgery

The use of VATS to treat both TCs and TNETs increased over time, rising from 1 (16.7%) in 2009 to 12 (48.0%) in 2018—a 31.3% increase over a 10-year period (Additional file 2: Fig. 2).

### Multivariable Cox proportional hazard analysis on the overall cohort

VATS was not found to be an independent predictive factor for TCs and TNETs in our Cox multiple regression analysis (hazard ratio [HR]: 0.46; 95% CI: 0.19–1.11; *P* = 0.084; Table 2). Age and Masaoka-Koga staging were determined to be independent predictive variables for TCs and TNETs.

**Table 2:** Independent predictors of survival in patients with thymic carcinomas and thymic neuroendocrine tumors adjusted for multivariable Cox proportional hazard analysis

**Variable**	**Hazard ratio**	**95% CI**	***P* value**
**VATS** (ref = open)	0.46	0.19, 1.11	0.084
**Age** (per year)	1.03	1.00, 1.06	0.041
**Male** (ref = female)	1.08	0.57, 2.04	0.819
**ECOG** (ref = 0)			
1	1.04	0.53, 2.06	0.905
2	2.18	0.92, 5.17	0.076
**Tumor size** (per cm)	0.97	0.85, 1.11	0.696
**Masaoka-Koga stage** (ref = stage I–IIA)			
IIB	1.41	0.26, 7.51	0.689
III	4.72	1.08, 20.64	0.039
IV	9.57	2.06, 44.42	0.004
**TNET** (ref = TC)	0.92	0.38, 2.23	0.857
**Length of stay(day), Median (IQR)**	1.00	0.96, 1.03	0.870
**level I care(day), Median (IQR)**	1.02	0.93, 1.12	0.708
**Intraoperative bleeding(ml), Median (IQR)**	1.00	1.00, 1.00	0.325

### Perioperative indicators and survival for open vs video-assisted thoracoscopic surgery in the overall cohort, before propensity score matching

Before PSM, 87 patients underwent open thoracotomy and 39 patients had VATS. Of the latter group, 13 had their VATS procedures changed to open ones, and were thus reallocated to the open chest group, based on the actual surgery that was performed. In terms of perioperative indicators, patients who underwent VATS scored better overall than those who underwent open surgeries; however, there was no difference between the two groups in terms of time spent in the postoperative intensive care unit (ICU). In terms of R0 resection rate, serious postoperative complications (including three cases of respiratory failure, three of pulmonary infection, two of chylothorax, two of cardiac arrhythmia, one of pulmonary air leak, and one of thrombosis), and 30- and 90-day mortality, there were no statistical differences between the VATS and open groups (Additional file 3: Table 1).

The median follow-up durations were 77 (95% CI: 64–97) and 57 (95% CI: 51–70) months in the open and VATS groups, respectively; and the 5-year survival rates before matching were not significantly different, at 54.5% and 67.8%, respectively (*P* = 0.062; Fig. 1C).

### Perioperative indicators and survival for open vs video-assisted thoracoscopic surgery in the overall cohort, after propensity score matching

After PSM, the open and VATS groups each had 27 patients. Population baseline characteristics were balanced between the groups (Table 1). The VATS group had a shorter length of hospital stay compared to the open group (13 vs. 16 days; *P* = 0.015), a shorter duration of Level I care, (0 vs. 1 day;* P* = 0.016) and less intraoperative bleeding (50 mL vs. 300 mL;* P* < 0.001). However, there were no significant differences in terms of surgical hospital cost, length of stay in the ICU, whether blood was transfused, infusion volume, and surgical duration in the matched population. Similarly, after matching, there were no significant differences between the two groups in terms of postoperative R0 resection rate, major postoperative complications, and both 30-day and 90-day mortality (Table 3).

**Table 3:** Perioperative indicators and short-term outcomes for open and thoracoscopic surgery after total population matching

**Variables**	**Total (*n* = 54)**	**Open (*n* = 27)**	**VATS (*n* = 27)**	***P***
**Length of stay(day), Median (IQR)**	14.0 (12.0, 17.8)	16.0 (13.0, 20.0)	13.0 (10.0, 15.0)	0.015
**Cost (CNY), Median (IQR)**	50580.0 (40330.0, 61870.0)	51900.0 (43880.0, 65390.0)	46310.0 (38040.0, 54660.0)	0.068
**ICU (day), Median (IQR)**	1.0 (1.0, 2.0)	1.0 (1.0, 2.0)	1.0 (1.0, 1.0)	0.224
**level I care(day), Median (IQR)**	0.0 (0.0, 1.0)	1.0 (0.0, 2.0)	0.0 (0.0, 0.0)	0.016
**Intraoperative bleeding(ml), Median (IQR)**	175.0 (50.0, 400.0)	300.0 (100.0, 600.0)	50.0 (45.0, 200.0)	< 0.001
**Intraoperative blood transfusion,*n* (%)**				0.111
No	50 (92.6)	23 (85.2)	27 (100)	
Yes	4 (7.4)	4 (14.8)	0 (0.0)	
**Infusion volume(ml), Median (IQR)**	1850.0 (1500.0, 2500.0)	2000.0 (1500.0, 2500.0)	1500.0 (1500.0, 2250.0)	0.083
**Operation duration(min), Median (IQR)**	183.0 ± 78.8	196.3 ± 75.9	169.6 ± 80.7	0.216
**R0 resection, n (%)**				1.000
Yes	46 (85.2)	23 (85.2)	23 (85.2)	
No	8 (14.8)	4 (14.8)	4 (14.8)	
**Postoperative complications, *n* (%)**				1.000
No	50 (92.6)	25 (92.6)	25 (92.6)	
Yes	4 (7.4)	2 (7.4)	2 (7.4)	
**30-Day mortality, *n* (%)**				1.000
No	54 (100.0)	27 (100.0)	27 (100.0)	
Yes	0 (0.0)	0 (0.0)	0 (0.0)	
**90-Day mortality, *n* (%)**				1.000
No	53 (98.1)	26 (96.3)	27 (100.0)	
Yes	1 (1.9)	1 (3.7)	0 (0.0)	

The median follow-up durations were 74 (95% CI: 57–99) months and 57 (95% CI: 51–70) months in the open and VATS groups, respectively; and the 5-year survival rates after matching were not significantly different, at 58.5% and 67.5%, respectively (*P* = 0.260; Fig. 1D).

### Subgroup analysis

Patients with stages I–IIIA malignancies who received VATS and open surgeries were matched in order to investigate variations in perioperative indicators and survival between the two. There were 73 patients in stages I–IIIA, with 17 in each of the matched VATS and open groups, and balanced baseline characteristics in both (Additional file 4: Table 2). After PSM, the VATS group had a shorter hospital stay, a length of Level I care, and less intraoperative bleeding. Similarly, there were no significant differences in R0 resection rates, major postoperative complications, or 30- and 90-day mortality rates between the two groups (Additional file 5: Table 3).

Long-term survival rates before matching were 66.4% and 69.4% in the open and VATS groups, respectively (*P* = 0.300; Fig. 1E), and remained statistically indistinguishable after matching, at 69.5% and 62.7%, respectively (*P* = 0.830; Fig. 1F).

### Frequency of transfer site distribution

**Fig. 2:**
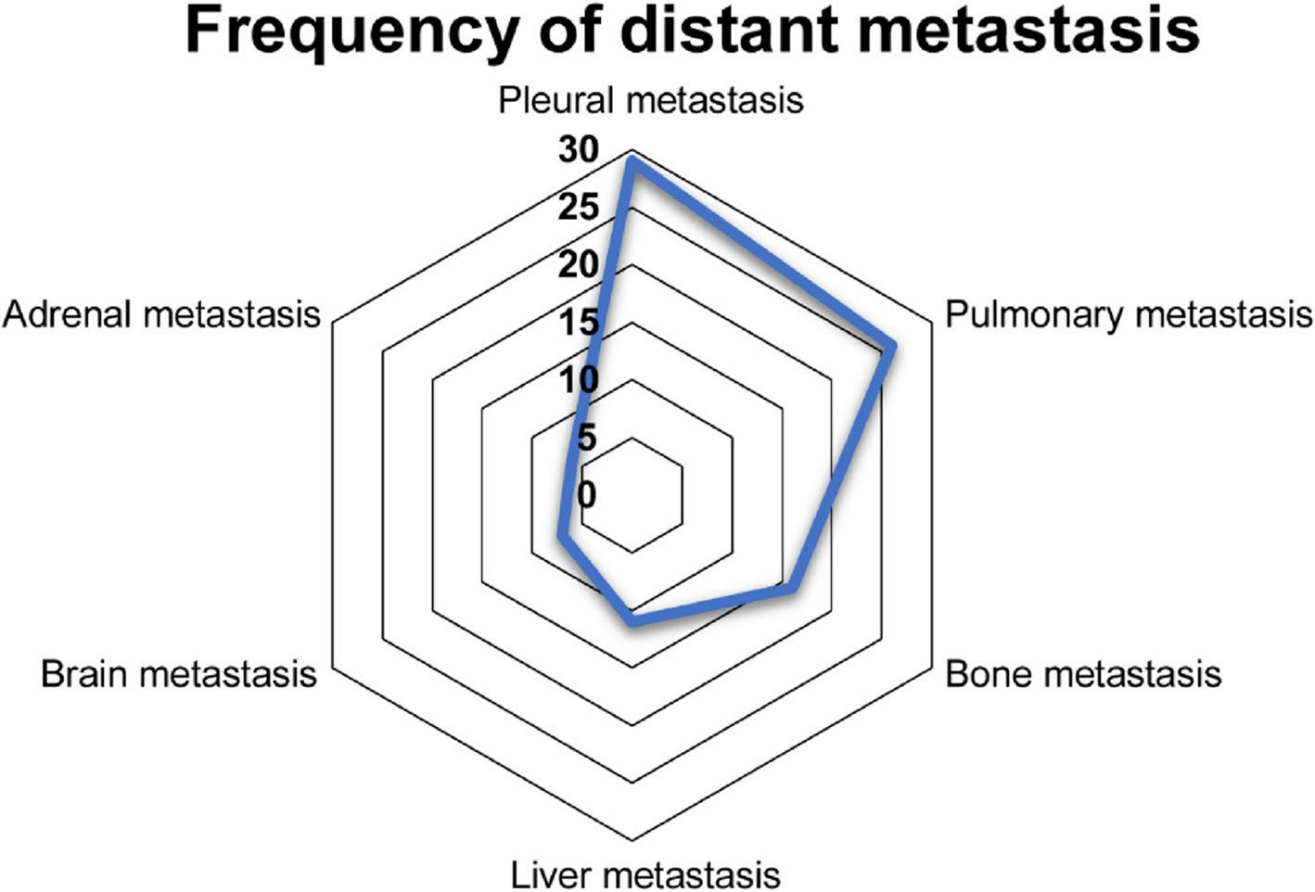
Frequency of transfer sites

The site and frequency of distant metastases of TCs and TNETs were counted to better investigate them. Pleural metastases were the most prevalent, with a total of 29, although pulmonary metastases were also common, with a total of 26. There were 16 cases of bone metastases, 11 cases of liver metastases, 7 cases of brain metastases, and 6 cases of adrenal metastases (Fig. 2).

## Discussion

Surgical methods have evolved rapidly over the past decades, and VATS is progressively being used with increasing frequency in many clinical practices. However, due to the rarity of TCs and TNETs, meaningful comparative surgical data are quite sparse for these particular malignancies. As a result, patients with thymomas have often been used as indirect evidence for these types of comparisons [13]. To date, three related studies have compared open-heart surgery to minimally-invasive surgery in patients with TC. In 2008, a study on eight cases of TCs in South Korea (four cases treated via endoscopy) only conducted a descriptive analysis, and preliminary results showed that endoscopic treatment seemed feasible in cases of early-stage TCs in Masaoka stages I and II. No related deaths or relapses were observed during the follow-up period, and the endoscopy group had less blood loss, pleural drainage, and shorter hospital stays [10]. Hurd et al. collected data from the National Cancer Database (NCDB) in the USA and found that, compared to open thymectomy, minimally invasive thymectomy(MIT) for stage I–III thymic cancer did not show significant differences in terms of short-term outcomes and overall survival. However, it is worth noting that there were only 22 patients with VATS in this cohort, and that, due to the limitations of the database, some important information such as surgical methods, diagnosis of *myasthenia gravis*, postoperative complications, and histological classification of thymic cancer, was missing. As well, 54.2% of the study cohort were patients with early-stage malignancies of Masaoka stages I–II, and a very small proportion were of Asian ethnicity [11]. Recently, Gu et al. focused on MIT for the treatment of T2-3NxM0 thymic malignancies, and their data suggested that it could be safely performed in carefully selected patients. Patients may benefit from minimally invasive surgery for better recovery, with comparable long-term oncological outcomes [12]. However, because the study included a specific stage population, and TCs and TNETs accounted for only 40% of the cases, only 23 cases of MIT were performed for TCs and TNETs, and their multifactorial analysis results showed that pathological type was the only independent factor that affected prognosis. The mortality risk ratio of TCs and TNETs was 3 × that of thymoma. Based on these points, it remains challenging to definitively answer specific questions regarding optimal surgical methods (i.e., VATS vs. open) for TCs and TNETs, and further relevant research is warranted to better address this important topic.

Our study included a larger sample size (39 patients who underwent VATS), in order to add to the body of work on this topic and thus provide better treatment options for TCs and TNETs in terms of improving patient prognoses and quality of life. We also collected more general information about the patients, such as insurance types, symptoms, presence of *myasthenia gravis*, ECOG scores, ACCI scores, pulmonary function, tumor size, Masaoka-Koga stage, pathology, chemotherapy, radiotherapy, immunotherapy, and more detailed perioperative related indicators, in order to increase the robustness of our analysis. This allowed us to balance the baseline characteristics between the two groups by statistical means such as PSM or multifactorial Cox regression, thus better comparing the prognostic and perioperative impacts of the two surgical interventions, which was then re-validated through a subgroup analysis.

The study comprised 126 patients with TCs and TNETs, with a median age of 56 years, most of whom were men. This is consistent with the findings of earlier studies, which have reported that these conditions occur mostly in male patients aged 54–65.5 years [14–17]. Because TCs and TNETs often infiltrate other tissues and are typically large at the time of diagnosis [17–20], patients frequently present with symptoms of compression of other organs, such as chest discomfort, coughing, and shortness of breath. Many other cases, however, are only discovered incidentally [21]. In our study 30.2% of the patients were seen for physical examinations without obvious clinical symptoms. In addition, TCs and TNETs are frequently seen in stages III–IV [15, 17], which accounted for 69.9% of the cases in our study. Adjuvant radiation treatment is strongly advised for patients with stage II–IVA TCs with positive surgical margins or full surgical resections, since it has been proven to improve both recurrence-free and overall survival [2, 22, 23]. This approach was taken for 71.4% of the patients in our study cohort. Platinum-based combination chemotherapy has also been used as part of a multimodal therapy for locally advanced TCs or TNETs [24], and 77.8% of the patients in our cohort received either neoadjuvant or adjuvant chemotherapy. PD-I checkpoint inhibitors, including pablizumab, have shown response rates of 19–23% for recurrent TCs [25, 26], and one subgroup of patients experienced lasting benefits from this approach, with median remission lengths ranging from 10 to 36 months. In our study, 12 patients received immunotherapy following recurrence or metastasis. The 5-year survival rate for the overall cohort was 58.5%, which is comparable with the 50–78% rates that have been reported in some previous studies [5, 6].

In our unadjusted and PSM analyses, we found no significant differences between the open and VATS groups in terms of short and long-term survival, although the VATS group showed better perioperative indicators. The same results were found in the subgroup with stage I–IIIA malignancies. Studies published in the preceding 5 years have reported that a higher age at diagnosis, lack of surgical treatment, and tumor stage are associated with shorter overall survival in patients with TC [27]. Age and stage were also found to be independent predictive variables for TCs and TNETs in our Cox multifactorial analysis. Previous research indicates that metastases from TCs most commonly occur in the pleura and lungs, but also in the liver, brain, extrathoracic lymph nodes, and adrenal glands, as well as in other organs [17, 28]. This is consistent with the location and frequency of metastases observed in our patients.

Our study does have some key limitations worth noting, the most significant of which is the small sample. However, to the best of our knowledge, this study currently has the largest number of VATS cases of any single-center study on TCs and TNETs. The retrospective study design also represents a limitation, as this may have led to a selective selection bias. To minimize these confounding factors, we applied PSM to maintain a balance between the two groups and thus determine the differences between the open and VATS groups. There are also certain patient-oriented indicators that are lacking, such as postoperative pain, care, and quality of life data. We did, however, gather tumor-related and perioperative indicators wherever feasible, in order to make our results more robust for comparison. Finally, due to the lengthy follow-up period, multi-center, large-scale patient data are warranted to corroborate our conclusions.

## Conclusions

This study found no statistically significant differences in 30-day and 90-day mortality or 5-year overall survival between patients who received either VATS and open surgeries to treat TCs and TNETs; however, the VATS group had better indicators in terms of total length of hospital stay, duration of Level I nursing, and bleeding, while maintaining comparable outcomes in the stage I–IIIA patient subgroup. This study supports the use of VATS for treating TCs and TNETs that have been determined to be entirely resectable following thorough examination by an experienced surgeon.

### Supplementary Information

**Additional file 1: Figure 1.** Flowchart.

**Additional file 2: Figure 2.** Changes in the surgical approach to thymic carcinoma and thymic neuroendocrine tumors over time.

**Additional file 3: Table 1.** Perioperative indicators and short-term outcomes for open and thoracoscopic surgery before total population matching.

**Additional file 4: Table 2.** Baseline information after matching for stage I-IIIA thymic carcinoma and thymic neuroendocrine tumors.

**Additional file 5: Table 3.** Perioperative indicators and short-term outcomes after matched open and thoracoscopic surgery for stage I-IIIA thymic carcinoma and thymic neuroendocrine tumors.

**Additional file 6: Table 4.** Summary of surgical accesses.

## Data Availability

The Second Affiliated Hospital of Air Force Military Medical University Hospital case system and imaging examination system are used to obtain all fundamental patient information and treatment data. After completing the application process with the hospital information section, our medical-related researchers will have access to hospital medical data files.

## Abbreviations

TETs: Thymic epithelial tumors
TCs: Thymic carcinomas
TNETs: Thymic neuroendocrine tumors
VATS: Video-assisted thoracoscopic surgery
PSM: Propensity score matching
ACCI: Age-adjusted Charlson Comorbidity Index
ECOG: Eastern Cooperative Oncology Group
IQR: Interquartile range
CI: Confidence interval
HR: Hazard ratio
ICU: Intensive care unit
MIT: Minimally invasive thymectomy
NCDB: National Cancer Database
