# Supplementary figures and images for: Effect of open versus video-assisted thoracoscopy on perioperative outcomes and survival for cases of thymic carcinomas and thymic neuroendocrine tumors
Source: World J Surg Oncol. 2023 Oct 16;21:329. doi: 10.1186/s12957-023-03210-7 (PMC10578011; doi:10.1186/s12957-023-03210-7)

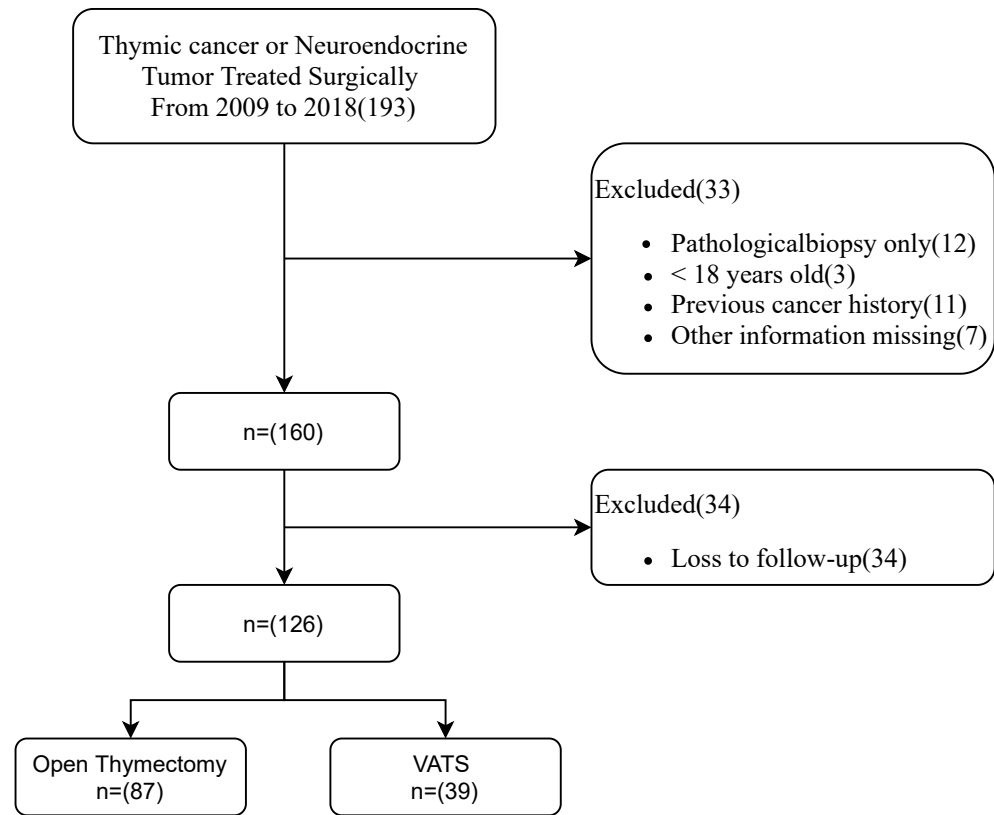

Supplement: Supplementary file 1 — Additional file 1: Figure 1. Flowchart. [file 12957_2023_3210_MOESM1_ESM.pdf]

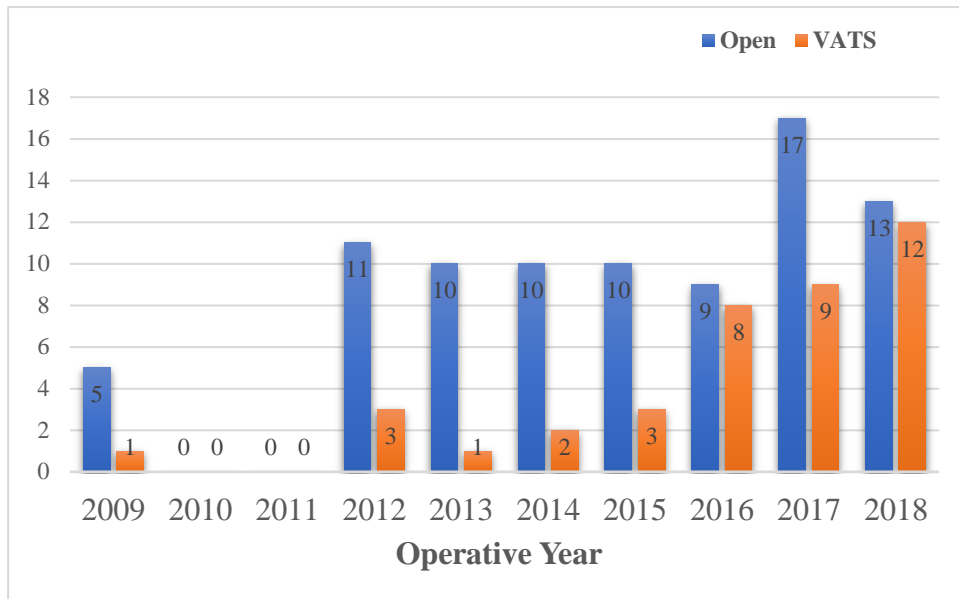

Supplement: Supplementary file 2 — Additional file 2: Figure 2. Changes in the surgical approach to thymic carcinoma and thymic neuroendocrine tumors over time. [file 12957_2023_3210_MOESM2_ESM.pdf]
