# Supplementary material for: Effect of open versus video-assisted thoracoscopy on perioperative outcomes and survival for cases of thymic carcinomas and thymic neuroendocrine tumors
Source: World J Surg Oncol. 2023 Oct 16;21:329. doi: 10.1186/s12957-023-03210-7 (PMC10578011; doi:10.1186/s12957-023-03210-7)
Supplement: Supplementary file 3 — Additional file 3: Table 1. Perioperative indicators and short-term outcomes for open and thoracoscopic surgery before total population matching. [file 12957_2023_3210_MOESM3_ESM.docx]

**Appendix Table1:** **Perioperative indicators and short-term outcomes for open and thoracoscopic surgery before total population matching**

|  |  |  |  |  |  |
| --- | --- | --- | --- | --- | --- |
| **Variables** | **Total** **(n = 126)** | **Open** **(n = 87)** | **VATS (n = 39)** | ***p*** | |
| **Length of stay(day), Median (IQR)** | 14.5 (11.0, 19.8) | 16.0 (12.0, 20.0) | 13.0 (9.0, 15.0) | < 0.001 | |
| **Cost (CNY), Median (IQR)** | 50980.0 (42140.0, 61090.0) | 53720.0 (45540.0, 63300.0) | 44120.0 (37740.0, 54660.0) | 0.002 | |
| **ICU (day), Median (IQR)** | 1.0 (1.0, 2.0) | 1.0 (1.0, 2.0) | 1.0 (1.0, 1.0) | 0.073 | |
| **level I care(day), Median (IQR)** | 0.0 (0.0, 2.0) | 1.0 (0.0, 3.0) | 0.0 (0.0, 0.0) | 0.001 | |
| **Intraoperative bleeding(ml), Median (IQR)** | 300.0 (100.0, 600.0) | 500.0 (200.0, 1200.0) | 50.0 (20.0, 125.0) | < 0.001 | |
| **Intraoperative blood transfusion, n (%)** |  |  |  | < 0.001 | |
| no | 92 (73.0) | 53 (60.9) | 39 (100) |  | |
| yes | 34 (27.0) | 34 (39.1) | 0 (0) |  | |
| **Infusion volume(ml), Median (IQR)** | 2000.0 (1500.0, 2500.0) | 2200.0 (1600.0, 2700.0) | 1500.0 (1500.0, 2000.0) | < 0.001 | |
| **Operation duration(min), Median (IQR)** | 180.0 (140.0, 240.0) | 210.0 (155.0, 260.0) | 150.0 (105.0, 185.0) | < 0.001 | |
| **R0 resection, n (%)** |  |  |  | 0.142 | |
| yes | 98 (77.8) | 64 (73.6) | 34 (87.2) |  | |
| no | 28 (22.2) | 23 (26.4) | 5 (12.8) |  | |
| **Postoperative complications, n (%)** |  |  |  | 0.753 | |
| no | 114 (90.5) | 78 (89.7) | 36 (92.3) |  | |
| yes | 12 (9.5) | 9 (10.3) | 3 (7.7) |  | |
| **30-Day mortality, n (%)** |  |  |  | 1.000 | |
| no | 124 (98.4) | 85 (97.7) | 39 (100) |  | |
| yes | 2 (1.6) | 2 (2.3) | 0 (0) |  | |
| **90-Day mortality, n (%)** |  |  |  | 0.310 | |
| no | 122 (96.8) | 83 (95.4) | 39 (100) |  | |
| yes | 4 (3.2) | 4 (4.6) | 0 (0) |  | |
|  |  |  |  |  | |
