# Supplementary material for: Effect of open versus video-assisted thoracoscopy on perioperative outcomes and survival for cases of thymic carcinomas and thymic neuroendocrine tumors
Source: World J Surg Oncol. 2023 Oct 16;21:329. doi: 10.1186/s12957-023-03210-7 (PMC10578011; doi:10.1186/s12957-023-03210-7)
Supplement: Supplementary file 4 — Additional file 4: Table 2. Baseline information after matching for stage I-IIIA thymic carcinoma and thymic neuroendocrine tumors. [file 12957_2023_3210_MOESM4_ESM.docx]

**Appendix Table 2: Baseline information after matching for stage I-IIIA thymic carcinoma and thymic neuroendocrine tumors**

|  | | |  |  | |  |  |
| --- | --- | --- | --- | --- | --- | --- | --- |
| **Variables** | **Total (n = 34)** | **Open (n = 17)** | | | **VATS (n = 17)** | | ***P*** |
| **Age, Median (IQR)** | 54.5 ± 11.9 | 52.7 ± 13.2 | | | 56.3 ± 10.6 | | 0.389 |
| **Sex, n (%)** |  |  | | |  | | 1.000 |
| female | 11 (32.4) | 6 (35.3) | | | 5 (29.4) | |  |
| male | 23 (67.6) | 11 (64.7) | | | 12 (70.6) | |  |
| **Insurance, n (%)** |  |  | | |  | | 0.766 |
| resident | 11 (32.4) | 6 (35.3) | | | 5 (29.4) | |  |
| employee | 9 (26.5) | 3 (17.6) | | | 6 (35.3) | |  |
| Self funded | 2 (5.9) | 1 (5.9) | | | 1 (5.9) | |  |
| non local | 12 (35.3) | 7 (41.2) | | | 5 (29.4) | |  |
| **Symptom, n (%)** |  |  | | |  | | 0.916 |
| physical examination | 13 (38.2) | 7 (41.2) | | | 6 (35.3) | |  |
| chest pain | 14 (41.2) | 6 (35.3) | | | 8 (47.1) | |  |
| respiratory symptoms | 4 (11.8) | 2 (11.8) | | | 2 (11.8) | |  |
| other compression symptoms | 3 (8.8) | 2 (11.8) | | | 1 (5.9) | |  |
| **Myasthenia gravis, n (%)** |  |  | | |  | | 1.000 |
| no | 34 (100.0) | 17 (100) | | | 17 (100) | |  |
| **ECOG, n (%)** |  |  | | |  | | 1.000 |
| 0 | 13 (38.2) | 7 (41.2) | | | 6 (35.3) | |  |
| 1 | 17 (50.0) | 8 (47.1) | | | 9 (52.9) | |  |
| 2 | 4 (11.8) | 2 (11.8) | | | 2 (11.8) | |  |
| **ACCI, n (%)** |  |  | | |  | | 0.102 |
| 0 | 9 (26.5) | 7 (41.2) | | | 2 (11.8) | |  |
| 1 | 10 (29.4) | 5 (29.4) | | | 5 (29.4) | |  |
| 2 | 7 (20.6) | 1 (5.9) | | | 6 (35.3) | |  |
| 3/3+ | 8 (23.5) | 4 (23.5) | | | 4 (23.5) | |  |
| **Pulmonary function, n (%)** |  |  | | |  | | 0.647 |
| normal | 19 (55.9) | 9 (52.9) | | | 10 (58.8) | |  |
| mild | 12 (35.3) | 6 (35.3) | | | 6 (35.3) | |  |
| moderate | 2 (5.9) | 2 (11.8) | | | 0 (0) | |  |
| severe | 1 (2.9) | 0 (0) | | | 1 (5.9) | |  |
| **Tumor size(cm), Median (IQR)** | 4.7 ± 1.7 | 4.7 ± 1.3 | | | 4.8 ± 2.1 | | 0.861 |
| **Masaoka-Koga stage, n (%)** |  |  | | |  | | 1.000 |
| I-IIA | 6 (17.6) | 3 (17.6) | | | 3 (17.6) | |  |
| IIB | 15 (44.1) | 7 (41.2) | | | 8 (47.1) | |  |
| IIIA | 13 (38.2) | 7 (41.2) | | | 6 (35.3) | |  |
| **Pathology, n (%)** |  |  | | |  | | 1.000 |
| TC | 29 (85.3) | 15 (88.2) | | | 14 (82.4) | |  |
| TNET | 5 (14.7) | 2 (11.8) | | | 3 (17.6) | |  |
| **Chemotherapy, n (%)** |  |  | | |  | | 0.707 |
| no | 10 (29.4) | 4 (23.5) | | | 6 (35.3) | |  |
| yes | 24 (70.6) | 13 (76.5) | | | 11 (64.7) | |  |
| **Radiotherapy, n (%)** |  |  | | |  | | 0.398 |
| no | 7 (20.6) | 2 (11.8) | | | 5 (29.4) | |  |
| yes | 27 (79.4) | 15 (88.2) | | | 12 (70.6) | |  |
| **Immunotherapy, n (%)** |  |  | | |  | | 1.000 |
| no | 32 (94.1) | 16 (94.1) | | | 16 (94.1) | |  |
| yes | 2 (5.9) | 1 (5.9) | | | 1 (5.9) | |  |
