# Supplementary material for: Effect of open versus video-assisted thoracoscopy on perioperative outcomes and survival for cases of thymic carcinomas and thymic neuroendocrine tumors
Source: World J Surg Oncol. 2023 Oct 16;21:329. doi: 10.1186/s12957-023-03210-7 (PMC10578011; doi:10.1186/s12957-023-03210-7)
Supplement: Supplementary file 5 — Additional file 5: Table 3. Perioperative indicators and short-term outcomes after matched open and thoracoscopic surgery for stage I-IIIA thymic carcinoma and thymic neuroendocrine tumors. [file 12957_2023_3210_MOESM5_ESM.docx]

**Appendix Table 3: Perioperative indicators and short-term outcomes after matched open and thoracoscopic surgery for stage I-IIIA thymic carcinoma and thymic neuroendocrine tumors**

| **Variables** | **Total (n = 34)** | **Open (n = 17)** | **VATS (n = 17)** | ***p*** |
| --- | --- | --- | --- | --- |
| **Length of stay(day), Median (IQR)** | 13.0 (10.2, 15.8) | 14.0 (12.0, 16.0) | 12.0 (9.0, 14.0) | 0.029 |
| **Cost (CNY), Median (IQR)** | 44590.0 (39810.0, 53890.0) | 46190.0 (43750.0, 55140.0) | 42570.0 (37860.0, 47510.0) | 0.235 |
| **ICU (day), Median (IQR)** | 1.0 (1.0, 1.0) | 1.0 (1.0, 2.0) | 1.0 (1.0, 1.0) | 0.124 |
| **level I care(day), Median (IQR)** | 0.0 (0.0, 2.8) | 1.0 (0.0, 3.0) | 0.0 (0.0, 0.0) | 0.035 |
| **Intraoperative bleeding(ml), Median (IQR)** | 100.0 (50.0, 300.0) | 200.0 (100.0, 400.0) | 50.0 (20.0, 200.0) | 0.011 |
| **Intraoperative blood transfusion, n (%)** |  |  |  | 0.485 |
| no | 32 (94.1) | 15 (88.2) | 17 (100) |  |
| yes | 2 (5.9) | 2 (11.8) | 0 (0) |  |
| **Infusion volume(ml), Median (IQR)** | 1500.0 (1500.0, 2000.0) | 1800.0 (1500.0, 2500.0) | 1500.0 (1000.0, 1700.0) | 0.058 |
| **Operation duration(min), Median (IQR)** | 150.0 (110.0, 206.2) | 155.0 (120.0, 240.0) | 140.0 (110.0, 180.0) | 0.617 |
| **R0 resection, n (%)** |  |  |  | 1.000 |
| yes | 34 (100.0) | 17 (100) | 17 (100) |  |
| **Postoperative complications, n (%)** |  |  |  | 1.000 |
| no | 32 (94.1) | 16 (94.1) | 16 (94.1) |  |
| **30-Day mortality, n (%)** |  |  |  | 1.000 |
| no | 2 (5.9) | 1 (5.9) | 1 (5.9) |  |
| **90-Day mortality, n (%)** |  |  |  | 1.000 |
| no | 34 (100.0) | 17 (100) | 17 (100) |  |
