# Supplementary material for: Effect of open versus video-assisted thoracoscopy on perioperative outcomes and survival for cases of thymic carcinomas and thymic neuroendocrine tumors
Source: World J Surg Oncol. 2023 Oct 16;21:329. doi: 10.1186/s12957-023-03210-7 (PMC10578011; doi:10.1186/s12957-023-03210-7)
Supplement: Supplementary file 6 — Additional file 6: Table 4. Summary of surgical accesses. [file 12957_2023_3210_MOESM6_ESM.docx]

**Appendix Table 4: Summary of surgical accesses.**

| Variables | Total (n = 126) | Open (n = 87) | VATS (n = 39) |
| --- | --- | --- | --- |
| Surgical approach, n (%) |  |  |  |
| sternotomy | 60 (47.6) | 60 (69.0) | 0 (0) |
| thoracotomy | 14 (11.1) | 14 (16.1) | 0 (0) |
| thoracoscopic to sternotomy | 7 ( 5.6) | 7 (8.0) | 0 (0) |
| thoracoscopic to thoracotomy | 6 ( 4.8) | 6 (6.9) | 0 (0) |
| thoracoscopic lateral approach | 13 (10.3) | 0 (0) | 13 (33.3) |
| thoracoscopic subxiphoid approach | 26 (20.6) | 0 (0) | 26 (66.7) |
